# Screening, identification, and experimental validation of SUMOylation biomarkers in Parkinson’s disease

**DOI:** 10.1186/s41065-025-00525-1

**Published:** 2025-08-08

**Authors:** Yifo Wei, Xinning Zhang, Rui Zuo, Wenxin Dang, Lu Chen, Fan Liu, Jia Yao, Weizheng Ran, Zhigang Chen, Xiaoyan Wang, Furong Lv, Yue Yu

**Affiliations:** 1 Xi’an Hospital of Traditional Chinese Medicine, Xi’an, China; 2 Shaanxi University of Chinese Medicine, Xianyang, China; 3 Dongfang Hospital, Beijing University of Chinese Medicine, Beijing, China; 4 The First Medical Center of PLA General Hospital, Beijing, China; 5 The Eighth Medical Center of PLA General Hospital, Beijing, China

**Keywords:** Parkinson's disease, SUMOylation, Biomarker, Machine learning, SUMO3, SEH1L

## Abstract

**Background:**

Parkinson’s disease (PD) is a common neurodegenerative disorder. The role of protein post-translational modifications (PTMs), especially small ubiquitin-like modifier (SUMO) conjugation (SUMOylation), in PD pathogenesis remains unclear. This study aimed to investigate the relationship between SUMOylation and PD.

**Methods:**

The analysis included the GSE22491 dataset, GSE18838 dataset, and 189 SUMO related genes. Differentially expressed genes (DEGs) between the PD group and the control group were identified in GSE22491; these were then intersected with SUMO related genes to identify candidate genes. Machine learning was used to select biomarkers consistent across both datasets, which were validated in GSE6631. Further analyses included back propagation (BP) neural network analysis, enrichment analysis, immune infiltration analysis, regulatory network construction, drug prediction, and molecular docking. Reverse transcription-quantitative polymerase chain reaction (RT-qPCR) was used to validate the biomarkers.

**Results:**

An overlap analysis of 3,222 DEGs and 189 SUMO related genes identified 25 candidate genes. Subsequent validation using the GSE22491 and GSE18838 datasets narrowed these biomarkers down to SUMO3 and SEH1L, which are involved in pathways (such as the nuclear pore pathway) associated with PD. Significant positive correlations were observed between specific immune cell subtypes and both biomarkers. Based on these correlations, relevant transcription factors (ZNF394, IRF4, FOXM1, EGR1) and drugs (Cianidanol, Methylmethanesulfonate, Valproic acid) were predicted. Additionally, RT-qPCR results confirmed that SUMO3 is significantly downregulated in PD.

**Conclusion:**

SUMO3 and SEH1L were identified as novel biomarkers for PD, offering potential targets for early diagnosis and therapy in PD.

**Supplementary Information:**

The online version contains supplementary material available at 10.1186/s41065-025-00525-1.

## Introduction

Parkinson’s disease (PD) is a progressive neurodegenerative disorder characterized by its continuous progression. Epidemiological data show that the global burden of PD has risen from 2.5 to 6.1 million cases over the past three decades [1]. In China, the prevalence of PD in the Han population aged ≥ 50 years is approximately 3.8756‰ [2]. With the aging population, it is projected that by 2030, the number of patients with PD in China will reach 4.94 million, accounting for nearly half of the global total [3]. While the majority of PD cases are sporadic, around 10% are familial. Numerous studies have identified associations between various genetic mutations and both sporadic and inherited forms of the disease [4]. Additionally, environmental exposures, such as pesticides and insecticides, are considered risk factors for PD, in conjunction with genetic factors [5]. The clinical manifestations of PD include motor symptoms such as bradykinesia, resting tremor, myotonia, and postural instability, as well as non-motor symptoms like hyposmia, autonomic dysfunction, sleep disturbances, depression, and cognitive decline [6]. Pathologically, PD is characterized by the degeneration and loss of dopaminergic neurons in the compact zone of the substantia nigra and the abnormal accumulation of α-synuclein (α-syn) into Lewy bodies in the remaining neurons [7]. Research into the pathogenesis of PD has demonstrated that inoculating α-syn fibrils [8] or α-syn oligomers extracted from the cerebrospinal fluid of patients with PD into the gastric wall or intestine induces the formation of phosphorylated α-syn positive aggregates in the dorsal motor nucleus of the vagus (DMV) and the Locus Coeruleus (LC) [9]. Furthermore, studies on α-syn-related structures have highlighted that α-syn oligomers play a pivotal role in the etiology of PD and may serve as key pathogenic seeds that trigger the abnormal aggregation and formation of Lewy bodies [10]. Despite significant research efforts, the precise etiology and pathogenesis of PD remain poorly understood. Current therapeutic strategies include pharmacological treatments, surgical interventions, exercise therapy, psychological support, and nursing care; however, no definitive cure exists [11]. Additionally, available clinical treatments only offer symptomatic relief without effectively halting disease progression [12]. Thus, the identification of reliable biomarkers for PD diagnosis and the advancement of research aimed at improving diagnostic and therapeutic approaches are imperative.

Protein post-translational modifications (PTMs) encompass chemical modifications of a protein’s structure and function occurring subsequent to translation, such as acetylation, glycosylation, phosphorylation, and methylation [13]. The Sentrin/small ubiquitin-like modifier (SUMO) pathway, a prevalent form of post-translational modification, regulates a variety of biological processes [14]. SUMO conjugation (SUMOylation), the covalent attachment of a SUMO to a target protein, plays a critical role in essential cellular functions including cell division, stress response, mitochondrial homeostasis, and DNA repair mechanisms [15]. Moreover, SUMOylation has been implicated in neurodegenerative diseases, such as Huntington’s disease (HD) [16]and Alzheimer’s disease (AD) [17]. In PD, SUMOylation facilitates the maintenance of aggregated α-synuclein in a soluble form [18]and modulates its extracellular release *via* vesicles [19]. Beyond α-synuclein, DJ-1, a protein involved in gene transcription and intracellular oxidative stress regulation, is also associated with PD. Mutations in the PARK7 gene encoding DJ-1 contribute to 1–2% of early-onset PD cases [20], with loss of DJ-1 function exacerbating disease progression [21]. Research suggests that enhancing DJ-1 SUMOylation may mitigate PD-related pathology [22]. In addition, SUMOylation can affect the stability and function of mitochondrial-related proteins [23], thereby influencing cellular energy metabolism and oxidative stress responses. Mitochondrial dysfunction is an important link in the pathogenesis of PD [24]. In contrast, although other post-translational modifications such as phosphorylation, acetylation, and ubiquitination also play important roles in cellular physiological processes, their direct association with the core pathological mechanisms of PD is relatively weaker. Currently, although there has been some research on the role of various post-translational modifications in neurodegenerative diseases, studies on SUMOylation in PD still have many gaps. Further exploration of the relationship between SUMOylation and PD is expected to fill the knowledge gaps in this field and provide new perspectives for understanding the pathogenesis of PD.

This study is the first to systematically analyze transcriptomic data from multiple public databases from the perspective of SUMOylation modification, integrating various machine learning methods, including LASSO regression, Support Vector Machine Recursive Feature Elimination (SVM-RFE), and the Boruta algorithm, to identify and validate SUMOylation-related biomarkers. Additionally, this study constructed a gene-drug interaction network and validated the binding capabilities of potential drugs to biomarkers through molecular docking experiments, providing new insights for the treatment of PD. Reverse transcription-quantitative polymerase chain reaction (RT-qPCR) was used to experimentally validate these biomarkers, further confirming their differential expression in PD. The findings offer a novel perspective for PD research and provide valuable references for clinical diagnosis.

## Methods

### Data collection

This study leveraged gene expression data from the GEO database (https://www.ncbi.nlm.nih.gov/gds). Specifically, the GSE22491 dataset (platform: GPL6480) included peripheral blood mononuclear cells (PBMC) from 10 patients with PD and 8 healthy controls, serving as the training set. The GSE18838 dataset (platform: GPL5175) contained peripheral blood from 17 patients with PD and 11 healthy controls, and the GSE6613 database (platform: GPL96) contained whole blood samples from 50 patients with PD and 23 healthy controls, which were used as a validation set. A total of 189 SUMO-RGs were retrieved from the Molecular Signatures Database (MSigDB) (https://www.gsea-msigdb.org/gsea/index.jsp) using the search term “reactome SUMOylation”.

### Difference expression analysis

Differential expression analysis between the PD and control groups in the GSE22491 dataset was performed using the limma package [25] to identify differentially expressed genes (DEGs), with the criteria set at P.adj < 0.05 and|log2 Fold Change (FC)| >0.5. Volcano plots were generated with the ggplot2 package [26] to visualize the DEG distribution, while heatmaps were created using the pheatmap package [27] to display DEG expression profiles.

### Recognition of feature genes

The VennDiagram package [28] was employed to intersect the DEGs and SUMO-RGs, yielding candidate genes for further analysis. Enrichment analysis was then conducted using the clusterProfiler package [29], focusing on Gene Ontology (GO) and Kyoto Encyclopedia of Genes and Genomes (KEGG) pathways (*P* < 0.05) to gain deeper insights into the biological functions and signaling pathways of the candidate genes. To explore potential interactions among these genes, a Protein-Protein Interaction (PPI) network was constructed using the STRING database (http://string.embl.de/) with a confidence threshold of 0.4. Clustering of the PPI network was performed using the MCODE plugin in Cytoscape [30], with module identification based on edge-node relationships. Threshold parameters (K-core = 2, degree cutoff = 2, max depth = 100, and node score cutoff = 0.2) were applied to define functional modules, with genes in these modules being recorded as feature genes.

### Machine learning

In order to construct a model that could effectively reduce the risk of overfitting and enhance the interpretability of the model, based on the GSE22491 dataset and feature genes, we used the glmnet software package [31] to perform LASSO logistic regression. We set the “family” parameter to “binomial” to conduct feature selection and weight adjustment. During the model construction process, we employed the method of 5 - fold cross - validation to determine the optimal model. Eventually, the LASSO regression selected Lambda.min as the best model. Lambda.min was the Lambda value that minimized the error of the model on the test set during the cross - validation process. The model corresponding to this value performed best in balancing model complexity and prediction accuracy. It could effectively avoid overfitting and ensure that the model had good prediction ability.

Furthermore, in order to further improve the reliability and generalization ability of the model and provide more accurate feature selection results, we applied the SVM–RFE method to the same feature genes and dataset. During the operation, we utilized the e1071 software package [32] to calculate the weights of each feature. Subsequently, we sorted the features according to their weights. Next, we carried out 5 - fold cross - validation to optimize the model and determine the optimal feature subset. It would be used for subsequent analysis and model building.

Similarly, to ensure comprehensive feature selection and reduce the risk of missing important features, we utilized the Boruta algorithm and the Boruta package [33] to select another set of genes from the feature genes in the GSE22491 dataset. Specifically, we executed the algorithm based on the Boruta function in the Boruta package. We set the number of model iterations to 100 to fully evaluate the importance of features and ensure the reliability and stability of the results. In each iteration, a random forest model was constructed to evaluate the original and shadow features and calculate the importance scores. Based on these scores, the algorithm classified the features into three categories: confirmed, tentative, and rejected. Meanwhile, we set an “importance threshold”. If the importance score of an original feature was significantly higher than the 95th percentile of the importance scores of the shadow features, the probability of it being labeled as “confirmed” increased. After 100 iterations of the algorithm, those features that were labeled as “confirmed” multiple times were finally identified as truly important features, thus forming the gene set selected by the Boruta algorithm.

Finally, the intersection of genes obtained from the three machine learning methods was used to derive candidate biomarkers. The cross - validation of multiple methods contributed to accurately determining the key genes, laying a foundation for subsequent analysis and modeling.

### Biomarker identification, diagnostic potential assessment, and chromosomal localization

The expression of candidate biomarkers was further assessed in the GSE22491 and GSE18838 datasets to determine their consistency and significance between PD and control groups. Biomarkers demonstrating consistent expression trends and significant differences (*P* < 0.05) across both datasets were selected for further analysis. And in the GSE6613 dataset, the rstatix package was used to further validate the expression of the screened biomarkers in the PD group and the normal control group through the Wilcoxon test analysis. To evaluate the diagnostic potential of these biomarkers, Receiver Operating Characteristic (ROC) curves were generated using the pROC package [34], and the area under the ROC curve (AUC) values were calculated for both the GSE22491, GSE18838 and GSE6613 datasets. Additionally, the chromosomal locations of the biomarkers were visualized using the RCircos package [35], with human chromosome data sourced from UCSC (UCSC.HG38.Human.CytoBandIdeogram) (https://genome.ucsc.edu/), creating circular plots to display the distribution of biomarkers across chromosomes.

### Functional analysis of biomarkers

A co-expression network for the biomarkers was constructed using GeneMANIA (http://www.genemania.org/), which identified additional genes associated with the functions of the biomarkers (fingerprint networks (FPD) < 0.05). To further validate the machine learning results, a Back propagation (BP) neural network was developed using the neuralnet package [36] for both the GSE22491 and GSE18838 datasets. ROC curves were again generated *via* the pROC package to assess the BP network’s ability to differentiate between PD and control samples. To explore the potential functions of the biomarkers, gene set enrichment analysis (GSEA) was performed in the GSE22491 dataset using the clusterProfiler package. This analysis calculated correlations between the biomarkers and other genes, followed by enrichment analysis based on an expression matrix sorted by correlation. The reference gene set c2.cp.kegg.v2023.1.Hs.symbols.gmt from the MSigDB database (https://www.gsea-msigdb.org/gsea/msigdb/) was utilized (P.adj < 0.05). The top 10 enriched pathways for each biomarker, along with the biomarkers themselves, were visualized in a gene-pathway network created in Cytoscape to investigate the gene-pathway interactions.

### Immune infiltration analysis

To explore the role of biomarkers in the immune microenvironment, the CIBERSORT algorithm was applied to the GSE22491 dataset to evaluate the content and relative abundance of immune cell types in both PD and control samples. The Wilcoxon test was used to assess differences between PD and control samples (*P* < 0.05). Additionally, Spearman correlation analysis was conducted to evaluate the relationship between biomarkers and differential immune cells (*P* < 0.05), with results visualized in heatmaps. Lastly, to examine the relationship between biomarkers and 17 immune-related pathways sourced from the ImmPort database (http://www.immport.org), Spearman correlation analysis was performed on all GSE22491 samples using the psych package [37](|cor| >0.3, *P* < 0.05).

### Cluster analysis and gene set variation analysis (GSVA) of clustering results

Consensus clustering of PD samples from the GSE22491 dataset was performed using the stats package [38] to investigate the relationship between biomarkers and PD subtypes based on expression patterns. The K-means clustering method, using Euclidean distance, was applied to classify the samples with the highest inter-group correlations and the lowest intra-group correlations. Principal component analysis (PCA) was then employed to assess the significance of the identified clusters. The Wilcoxon test was used to compare biomarker expression across the different PD subtypes (*P* < 0.05). Gene Set Variation Analysis (GSVA) was employed to examine pathway enrichment differences between PD subtypes, focusing on biological pathway discrepancies among biomarker-defined subtypes. The gene set c2.cp.kegg.v2022.1.Hs.symbols.gmt, which includes metabolic pathways, was input into GSVA to calculate sample-specific gene set scores. Significant pathway variations across subtypes were determined using the Wilcoxon test (*P* < 0.05). Pathways were classified as activated in the TYPE2 group (t > 0) or the TYPE1 group (t < 0).

### Regulatory network construction

To explore the regulatory mechanisms of biomarkers, transcription factors (TFs) involved in biomarker regulation were predicted using chip-seq data from the ENCODE database through the NetworkAnalyst online platform (http://www.networkanalyst.ca/). Additionally, target microRNAs (miRNAs) implicated in biomarker regulation were forecasted using the starBase database (http://starbase.sysu.edu.cn/) (clipExpNum > 5). Interactions between biomarkers and PD-related diseases were examined using the DisGeNET database (https://www.disgenet.org/). The results were visualized using Cytoscape software.

### Drug prediction and molecular docking

For the identification of potential therapeutic targets for PD, biomarkers were queried in the Database of Signatures (DSigDB) (dsigdb.tanlab.org) to predict drugs that might interact with them, and the results were represented in a drug-gene network. The drugs predicted by multiple biomarkers were subjected to molecular docking. The process involved retrieving the protein structures of the biomarkers from the Protein Data Bank (PDB) (https://www.rcsb.org), stripping small molecules and water, and preparing the structures for docking by adding hydrogens and calculating charges using AutoDock Tools. Active ingredients from PubChem (https://pubchem.ncbi.nlm.nih.gov) were also prepared, ensuring charge balance and checking for rotatable bonds. The docking box was defined based on the receptor’s active site, and receptor-ligand docking was conducted using AutoDock Vina [39]. The docking box parameters were set with the protein’s active pocket as the center, and the search space was determined based on the receptor structure. For the search parameters, “exhaustiveness” was set to 32 to enhance the depth of conformational sampling, and the top 20 binding conformations were output. The conformation with the lowest binding free energy (ΔG, in kcal/mol) was selected as the optimal result, and a binding energy of ≤ -4.0 kcal/mol indicated strong binding activity. Visualization and aesthetic enhancement of the results were carried out using PyMol [40].

### RT-qPCR

RT-qPCR validation of biomarkers was performed using whole blood samples from 5 patients with PD and 5 control subjects, with the RT-qPCR experiments approved by Xi’an Hospital of Traditional Chinese Medicine (approval number: LLSCPJ- 2021007), All patients provided written informed consent. Total RNA was extracted from control and PD samples using TRIzol reagent. RNA quality was assessed using the Nanodrop N50 spectrophotometer, and the RNA was reverse-transcribed into cDNA using the SweScript First Strand cDNA synthesis kit (Servicebio, China). The cDNA was subsequently subjected to qPCR according to the manufacturer’s instructions. The 2-△△CT method was used to calculate relative gene expression levels, with GAPDH as the internal reference gene.In addition, the primers used for the experiments were from Tsingke Biotech (Beijing, China), and their detailed sequences are listed in Supplementary Table 1.

### Statistical analysis

All statistical analyses were performed using R, and the version number of the R package used is listed in the Supplementary Table 2. P-values < 0.05 were considered statistically significant unless otherwise stated,.

## Results

### The 7 feature genes were associated with multiple biological pathways

**Fig. 1:**
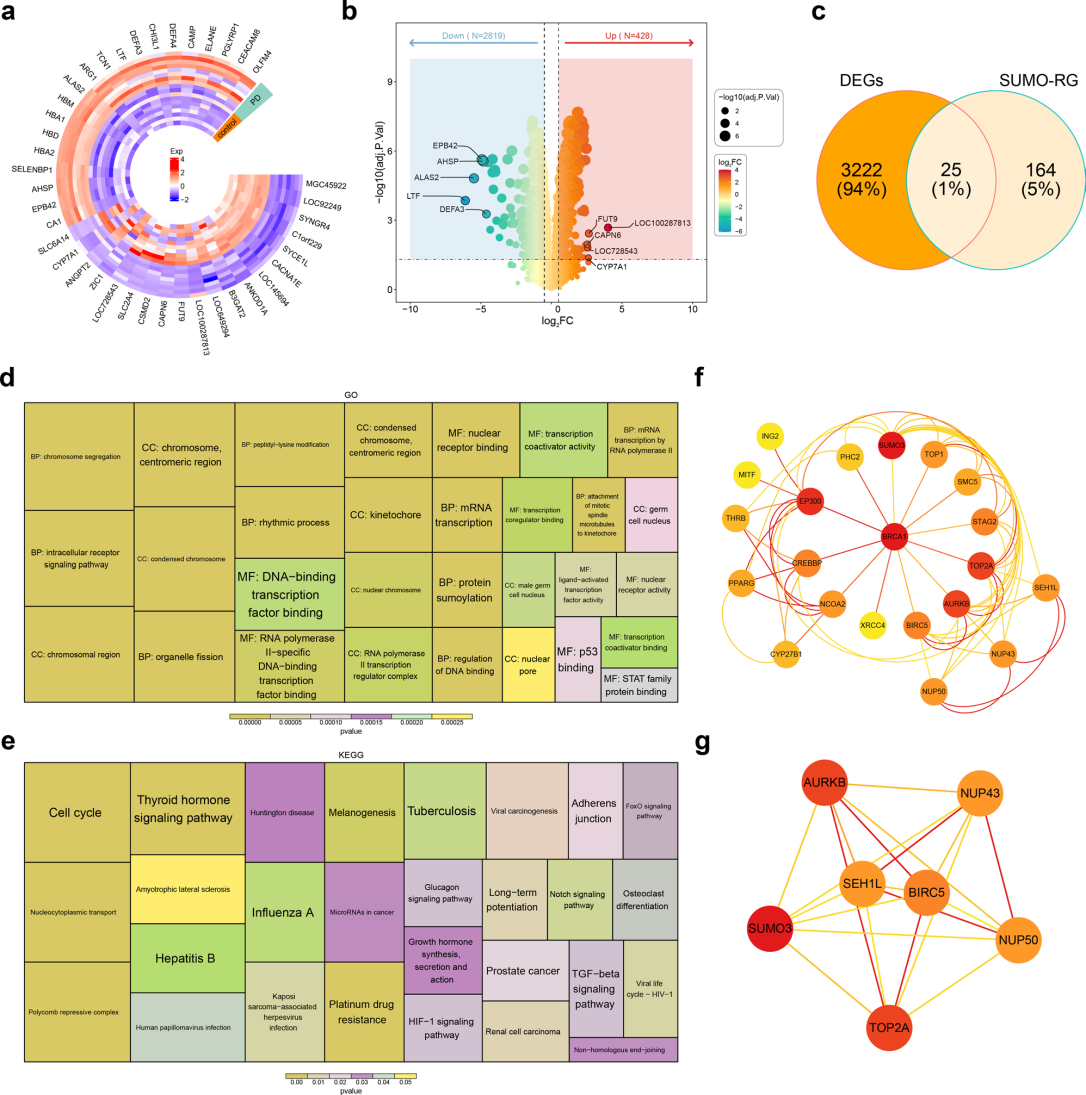
Differential expression analysis between PD and control samples. (**A**) Volcano plot of DEGs between the PD and control samples. The left blue area represents downregulated genes; the right red area represents upregulated genes. (**B**) Heatmap of DEGs between the PD and control samples. Red represents high expression, blue represents low expression. (**C**) Venn diagram of DEGs and 189 SUMO-RGs. (**D**) The result of GO enrichment. (**E**) The result of KEGG enrichment. (**F**) A PPI network. (**G**) Selection of feature genes used the MCODE plugin in Cytoscape. PD, Parkinson’s disease; DEGs, differentially expressed genes; SUMO-RGs, SUMOylation related genes; GO, Gene Ontology; KEGG, Kyoto Encyclopedia of Genes and Genomes; PPI, Protein-Protein Interaction

Differential expression analysis identified 3,247 DEGs between PD and control samples, including 428 up-regulated and 2,819 down-regulated genes (Fig. 1A-B). The intersection of these DEGs with 189 SUMO-RGs yielded 25 candidate genes (Fig. 1C). Enrichment analysis of these candidate genes revealed significant associations with 669 GO terms, comprising 42 Cellular Components (CCs), 57 Molecular Functions (MFs), and 570 Biological Processes (BPs), including condensed chromosome and peptidyl-lysine modification (Fig. 1D). Additionally, the candidate genes were linked to 28 KEGG pathways, such as the polycomb repressive complex, nucleocytoplasmic transport, and the cell cycle (Fig. 1E). A PPI network, constructed with 21 nodes and 65 edges, revealed key relationships, including BRCA1-TOP2A and SUMO3-TOP1 (Fig. 1F). Using the MCODE plugin in Cytoscape, seven feature genes were identified: AURKB, NUP43, SUMO3, TOP2A, NUP50, SEH1L, and BIRC5 (Fig. 1G).

### SUMO3 and SEH1L were pinpointed as potential biomarkers

**Fig. 2:**
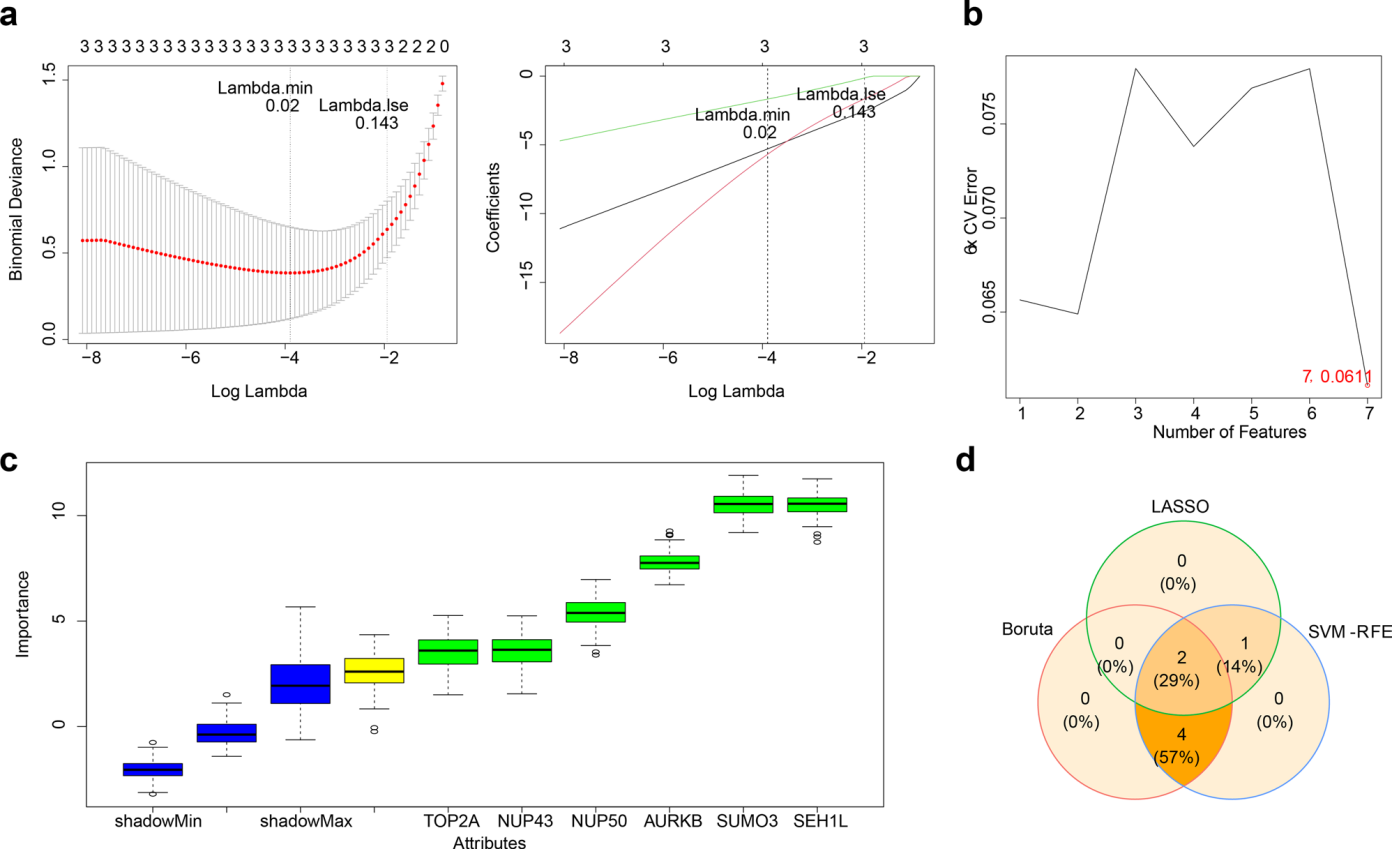
Identification of candidate biomarkers. (**A**) LASSO regression model for potential biomarkers selection. (**B**) SVM-RFE method algorithm for potential biomarkers selection, with 6-fold cross-validation. (**C**) Boruta algorithm for potential biomarkers selection. (**D**) Venn diagram of potential biomarkers identified by LASSO, SVM-RFE, and Boruta

The LASSO regression selected Lambda.min as the best model. The Measure was 0.3845 and the SE was 0.265, indicating that the performance of the model was good. In the LASSO regression model, a lambda.min value of approaching 0.02 selected three genes: SUMO3, SEH1L, and BIRC5 (Fig. 2A). The SVM-RFE method, with 5-fold cross-validation at 0.0611, identified seven genes: AURKB, NUP43, SUMO3, TOP2A, NUP50, SEH1L, and BIRC5 (Fig. 2B; Table 1). The Boruta algorithm also identified six genes: TOP2A, AURKB, NUP43, SUMO3, NUP50, and SEH1L (Fig. 2C). The intersection of these three gene sets led to the identification of two final candidate biomarkers: SUMO3 and SEH1L (Fig. 2D).

**Table 1:** The potential biomarkers identified by SVM-RFE

Feature Name	Feature ID	Avg Rank
SEH1L	6	1.5
SUMO3	3	2.0
BIRC5	7	3.5
NUP43	2	4.3
NUP50	5	5.2
AURKB	1	5.7
TOP2A	4	5.8

Note: SVM-RFE, Support Vector Machine-Recursive Feature Elimination

### SUMO3 and SEH1L were identified as biomarkers

**Fig. 3:**
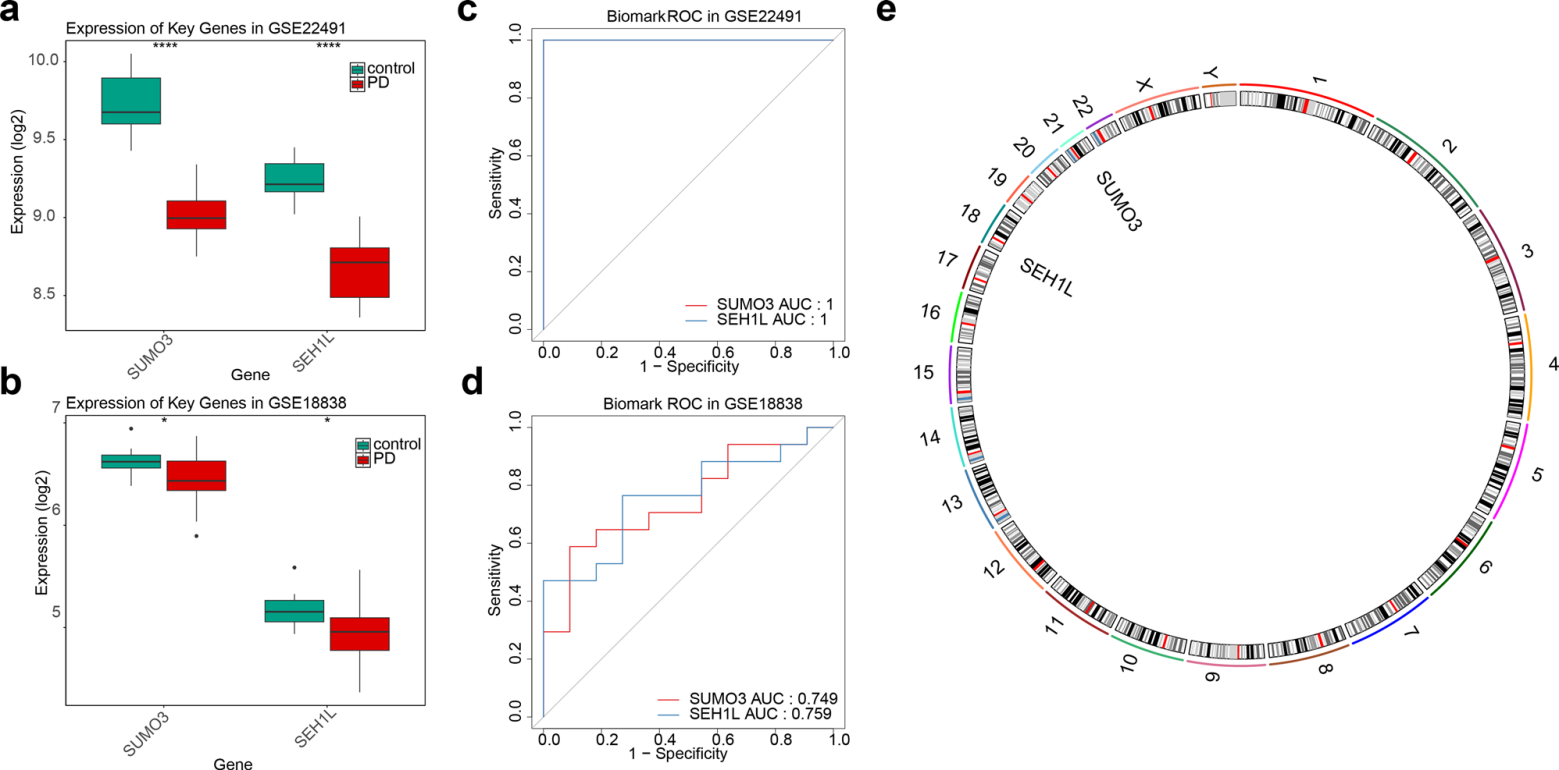
Expression analysis, ROC curve, and chromosomal localization of SUMO3 and SEH1L. (**A**-**B**) The expression of SUMO3 and SEH1L in GSE22491 and GSE18838 datasets. * *p* < 0.05, ** *p* < 0.01, and *** *p* < 0.001. (**C**-**D**) The ROC curves of SUMO3 and SEH1L in GSE22491 and GSE18838 datasets. (**E**) Chromosomal localization analysis for SUMO3 and SEH1L. ROC, Receiver Operating Characteristic

The expression of SUMO3 and SEH1L was assessed in both the GSE22491 and GSE18838 datasets. Consistent expression patterns and significant down-regulation in PD samples compared to controls were observed for both biomarkers in both datasets (Fig. 3A-B). Meanwhile, in the whole blood dataset GSE6613, the trends of two genes, SUMO3 and SEH1L, were consistent with those in the training set. Both genes were down-regulated and the down-regulation was significant (Supplementary Fig. 1). As a result, SUMO3 and SEH1L were identified as potential biomarkers for PD. ROC curve analysis revealed an AUC greater than 0.7 for both biomarkers in both datasets, indicating their strong potential to discriminate between PD and control groups (Fig. 3C-D). Subsequently, the model’s prediction results were evaluated using a ROC curve. In the GSE6613 dataset, the AUCs of the two biomarkers were both greater than 0.63, indicating that they had good discrimination potential between the PD group and the control group (Supplementary Fig. 2). Chromosomal localization analysis revealed that SUMO3 is located on chromosome 21, while SEH1L is located on chromosome 18 (Fig. 3E).

### SUMO3 and SEH1L shared common biological functions in PD

**Fig. 4:**
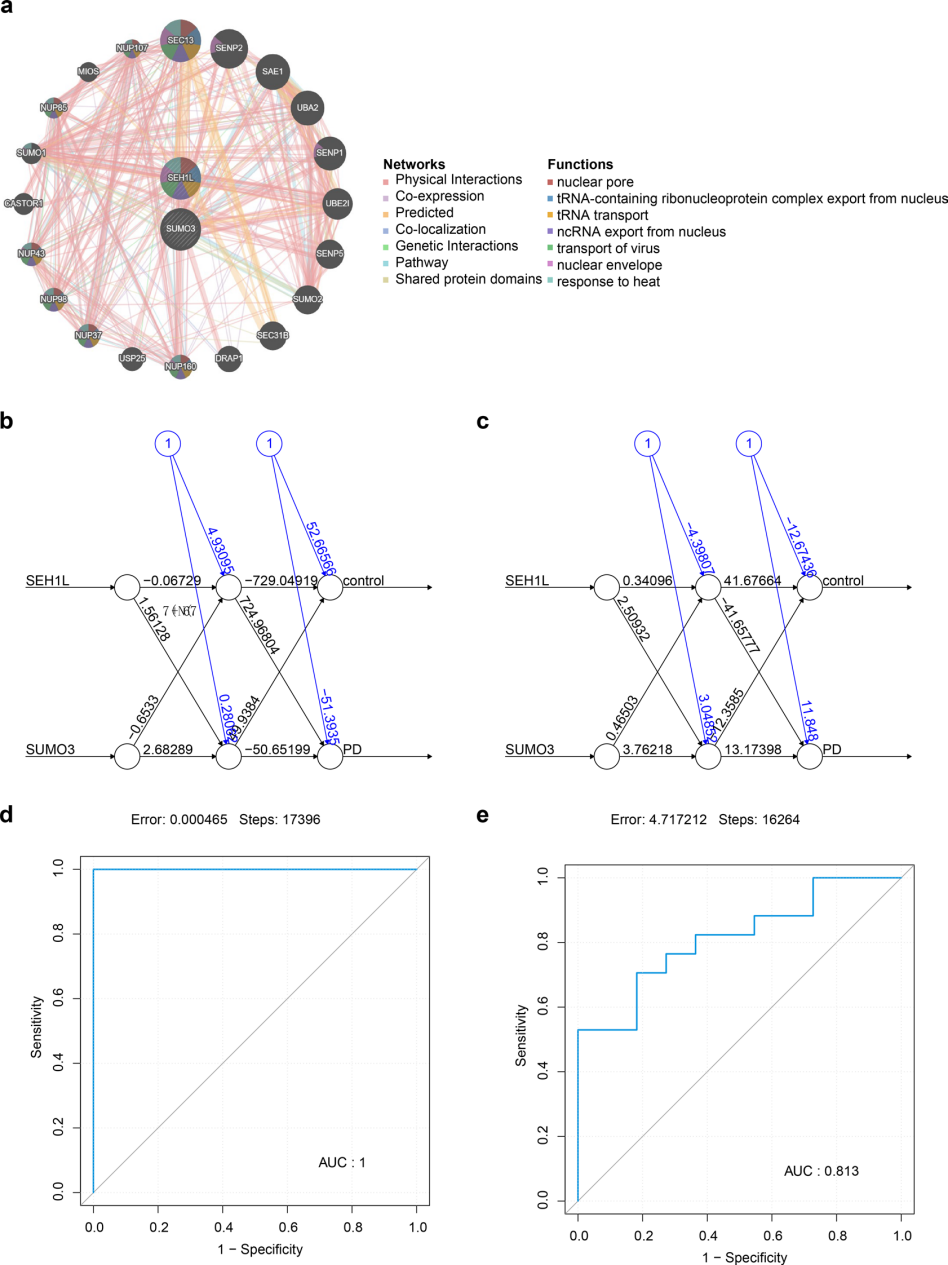
GGI network and BP neural network. (**A**) GGI network. (**B**-**C**) Construction of a BP neural network in GSE22491 and GSE18838 datasets. (**D**-**E**) The ROC curves of the BP neural network in GSE22491 and GSE18838 datasets. GGI: Gene-Gene Interaction; BP, Backpropagation Neural

A network analysis predicted 20 genes with the strongest functional similarity to SUMO3 and SEH1L, highlighting numerous interactions, such as SUMO3-UBA2 and SEH1L-SUMO1 (Fig. 4A). These genes were primarily involved in nuclear pore function, tRNA transport, virus transport, and ncRNA export from the nucleus. Furthermore, a BP neural network was constructed using the GSE22491 and GSE18838 datasets to assess the combined ability of SUMO3 and SEH1L to distinguish between PD and control samples (Fig. 4B-C). The AUC values of the BP neural network exceeded 0.7 in both datasets, demonstrating the network’s effective discriminatory power between PD and control groups (Fig. 4D-E).

### SUMO3 and SEH1L were co-enriched in Snca to 26s proteasome-mediated protein degradation

**Fig. 5:**
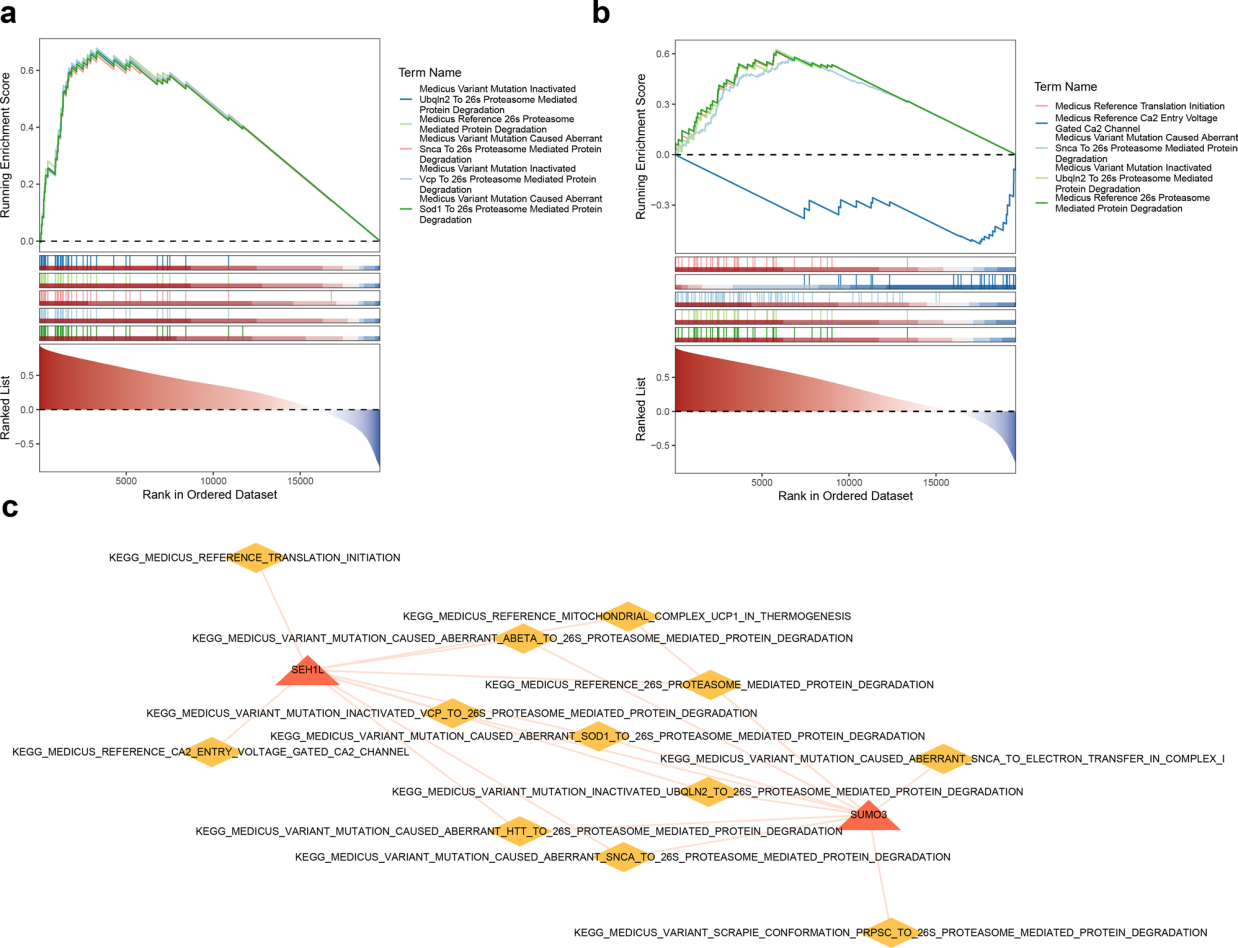
GSEA and gene-pathway network. (**A**-**B**) The GSEA results of SUMO3 and SEH1L. Divided into three parts. At the top is the ES value curve, and the highest/lowest point score corresponds to the ES value of the pathway. ES is positive, indicating that positively correlated genes dominate the pathway. The middle represents the position of pathway genes in the sorted list, with vertical lines representing genes and colors ranging from red to blue, indicating positive to negative correlations. At the bottom is the distribution chart of correlation coefficients. (**C**) A gene-pathway network constructed from the top 10 enriched pathways for each biomarker. The red nodes represent biomarkers, and the yellow nodes represent pathways. GSEA, Gene Set Enrichment Analysis

GSEA was conducted to investigate the signaling pathways associated with SUMO3 and SEH1L. The results demonstrated that both SUMO3 and SEH1L were co-enriched in the pathway from snca to 26 S proteasome-mediated protein degradation (Fig. 5A-B). Additionally, SUMO3 showed enrichment in several other pathways, including sod1 to 26 S proteasome-mediated protein degradation and vcp to 26 S proteasome-mediated protein degradation. In contrast, SEH1L was significantly associated with pathways such as the Medicus reference translation initiation and Medicus reference Ca²⁺ entry through voltage-gated Ca²⁺ channels. These results suggest that SUMO3 and SEH1L may play key roles in the physiological processes of protein degradation in PD. A gene-pathway network was subsequently constructed based on the top 10 enriched pathways for each biomarker, featuring 14 nodes and 20 edges, and highlighting key relationships such as SEH1L-Medicus reference translation initiation and SUMO3-Medicus reference mitochondrial complex UCP1 in thermogenesis (Fig. 5C).

### SUMO3 and SEH1L showed consistent correlations with differential immune cells

**Fig. 6:**
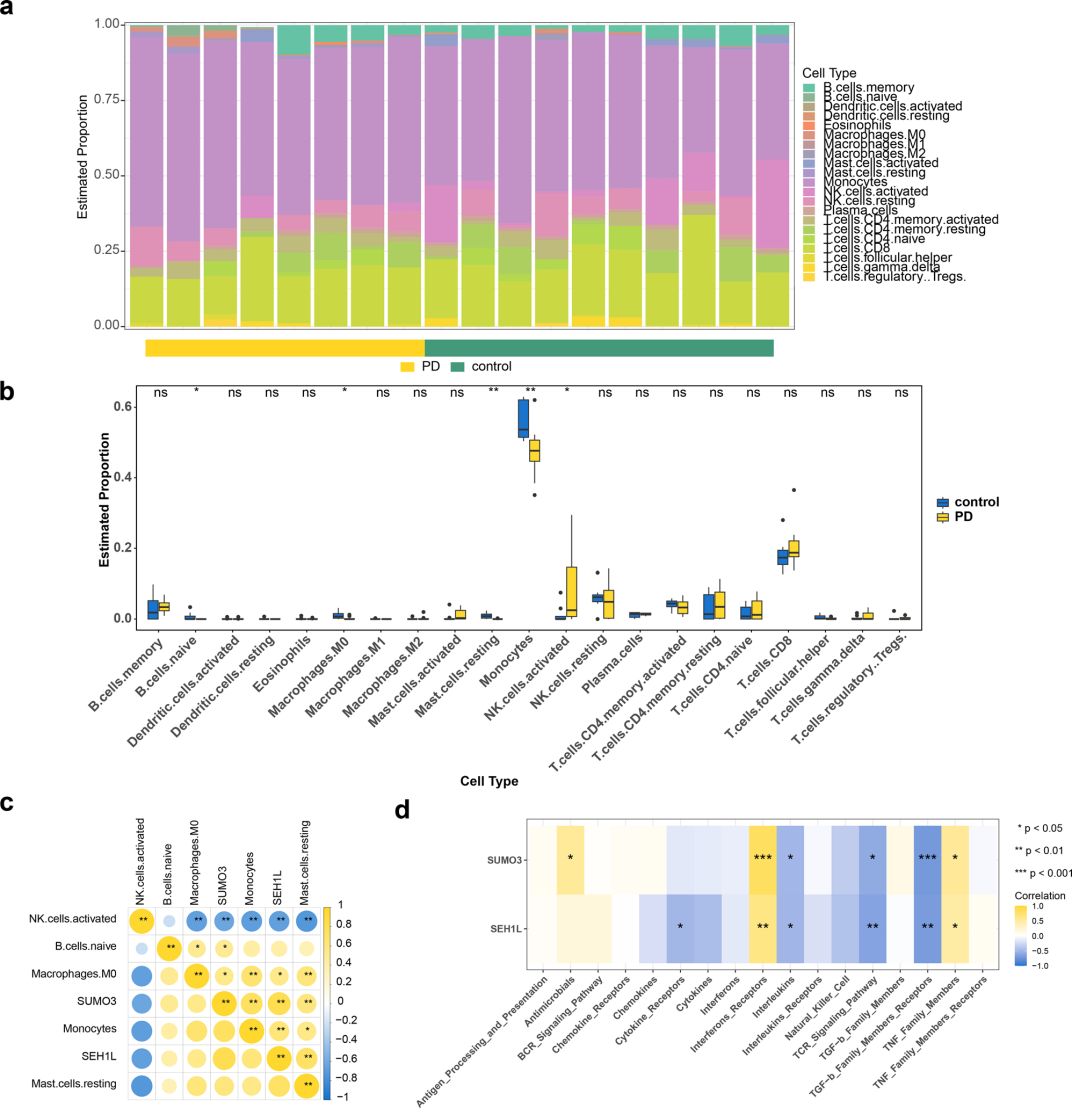
Immune infiltration analysis. (**A**) The infiltration abundance of immune cells in GSE22491. (**B**) Differences in infiltration levels of immune cell types between PD and the control samples. * *p* < 0.05, ** *p* < 0.01, and *** *p* < 0.001. (**C**) The correlations between the biomarkers and differential immune cells. Yellow represents positive correlation, blue represents negative correlation. The darker the color, the stronger the correlation. * *p* < 0.05, ** *p* < 0.01, and *** *p* < 0.001. (**D**) The correlations between the biomarkers and 17 immune-related pathways. Yellow represents positive correlation, blue represents negative correlation. * *p* < 0.05, ** *p* < 0.01, and *** *p* < 0.001. PD, Parkinson’s disease

Further analysis of immune cell infiltration in the GSE22491 dataset revealed differences in the immune cell composition between PD and control groups (Fig. 6A). Five immune cell types exhibited differential infiltration levels: naive B cells, resting M0 macrophages, resting mast cells, monocytes, and activated natural killer (NK) cells (Fig. 6B). Notably, NK cells showed a significantly higher infiltration score in the PD group, while other immune cell types displayed contrasting results. Correlation analysis indicated that SUMO3 and SEH1L were consistently correlated with the differential immune cell populations. Significant positive correlations were observed for most immune cells, except for activated NK cells (*P* < 0.05) (Fig. 6C). Specifically, SUMO3 exhibited the strongest positive correlation with monocytes (cor = 0.75, *P* < 0.05), while SEH1L demonstrated the most pronounced negative correlation with activated NK cells (cor = -0.67, *P* < 0.01). In addition, when analyzing the relationship between the biomarkers and 17 immune-related pathways, SUMO3 showed the strongest positive correlation with interferon receptors (cor = 0.81, *P* < 0.001) and the strongest negative correlation with TGF-β family member receptors (cor = -0.71, *P* < 0.001) (Fig. 6D).

### The biomarkers were significantly overexpressed in the type1 subtype

**Fig. 7:**
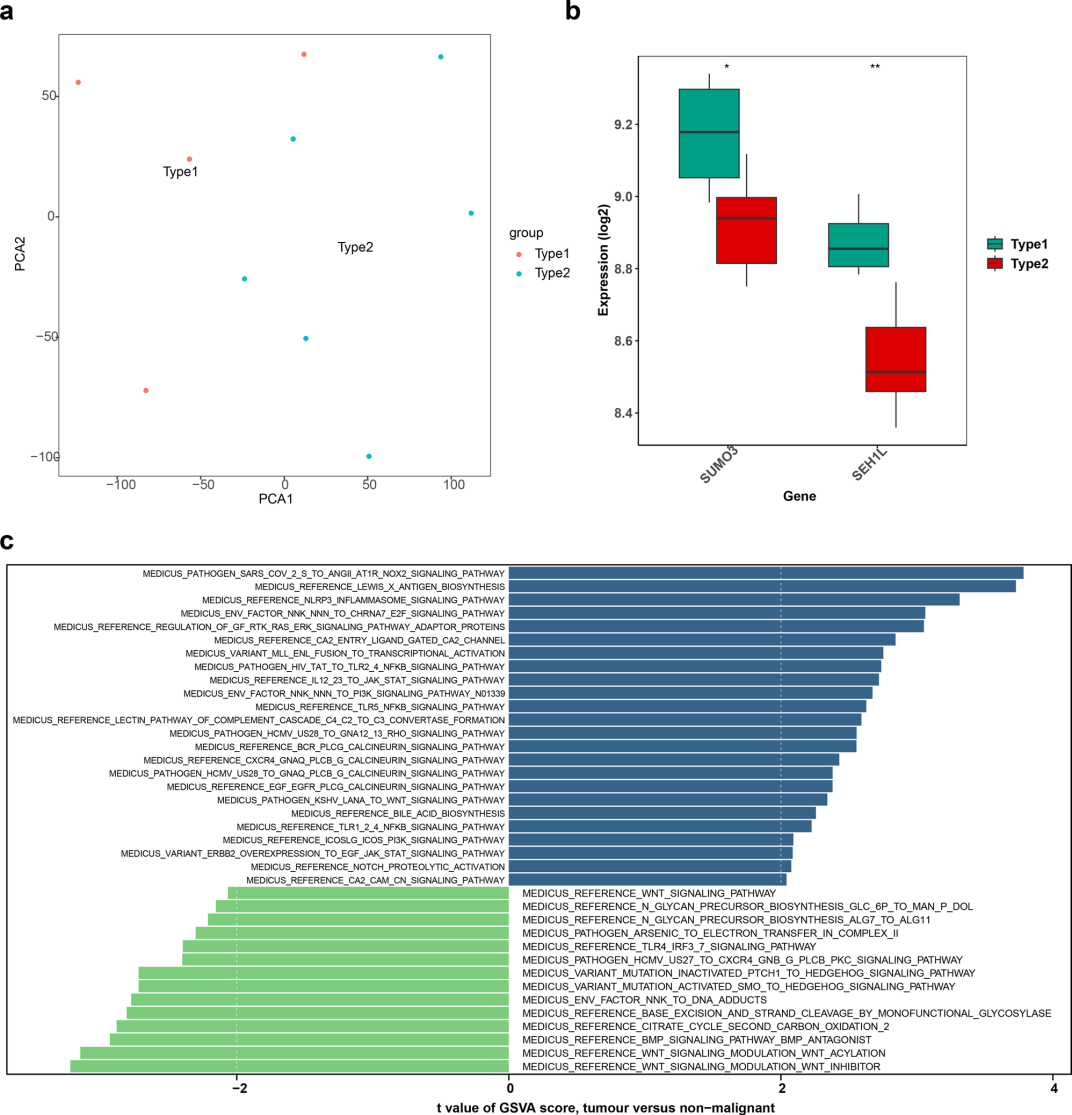
K-means clustering analysis. (**A**) PCA plot of type 1 and type 2. (**B**) The expression differences of biomarkers between type 1 and type 2. * *p* < 0.05, ** *p* < 0.01, and *** *p* < 0.001. (C) GSVA results between type 1 and type 2. Each row is a pathway, abscissa is t-value. Green (t < 0) pathways were activated in high-risk group; blue (t > 0) pathways were activated in low-risk group. PCA, Principal Component Analysis; GSVA, Gene Set Variation Analysis

K-means clustering (Fig. 7A) of PD samples from the GSE22491 dataset revealed two distinct subgroups (Type 1 and Type 2), as confirmed by PCA, which showed clear separation between these two subtypes. Biomarker expression was significantly higher in the Type 1 subtype (*P* < 0.05) (Fig. 7B). Pathway enrichment analysis of PD subtypes revealed that 20 pathways, including Lewis X antigen biosynthesis and IL12/23 to JAK-STAT signaling, were activated in Type 2, while 12 pathways, such as Wnt signaling modulation (both Wnt inhibitor and Wnt acylation), were activated in Type 1 (Fig. 7C).

### The biomarkers were associated with multiple factors and various diseases

**Fig. 8:**
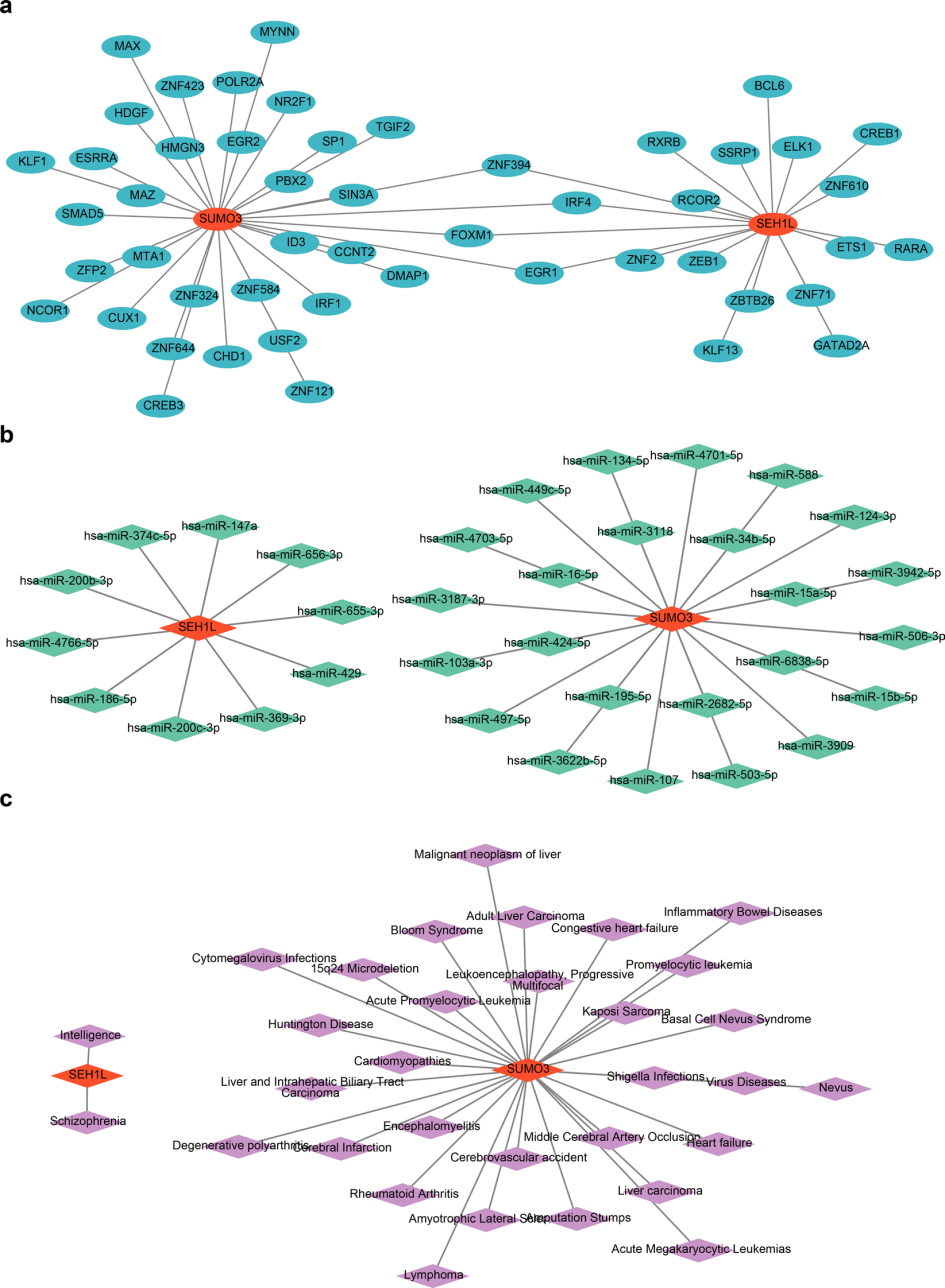
Construction of regulatory networks. (**A**) Gene-TF regulatory network. The red nodes represent biomarkers, and the blue nodes represent TFs. (**B**) Gene-miRNA regulatory network. The red nodes represent biomarkers, and the green nodes represent miRNAs. (**C**) Gene-disease regulatory network. The red nodes represent biomarkers, and the purple nodes represent diseases

A total of 35 TFs were predicted for SUMO3, while 9 TFs were predicted for SEH1L. A regulatory network was constructed to illustrate the interactions between these TFs and the biomarkers, containing 52 nodes and 54 edges. The interaction pairs identified in the network included SUMO3-SIN3A and SEH1L-ZNF71 (Fig. 8A). Notably, ZNF394, IRF4, FOXM1, and EGR1 were predicted as common TFs for both SUMO3 and SEH1L. In addition, miRNA predictions were also made, where 24 miRNAs were predicted for SUMO3 and 10 for SEH1L, resulting in the construction of a gene-miRNA regulatory network with 52 nodes and 54 edges (Fig. 8B). The network revealed key interaction pairs such as SEH1L-hsa-miR-429 and SUMO3-hsa-miR-6838-5p. Furthermore, the biomarkers were linked to various diseases, including schizophrenia, encephalomyelitis, and Huntington’s disease (Fig. 8C).

### Drug-biomarker interaction network construction and molecular docking analysis revealed dual-targeting drug

**Fig. 9:**
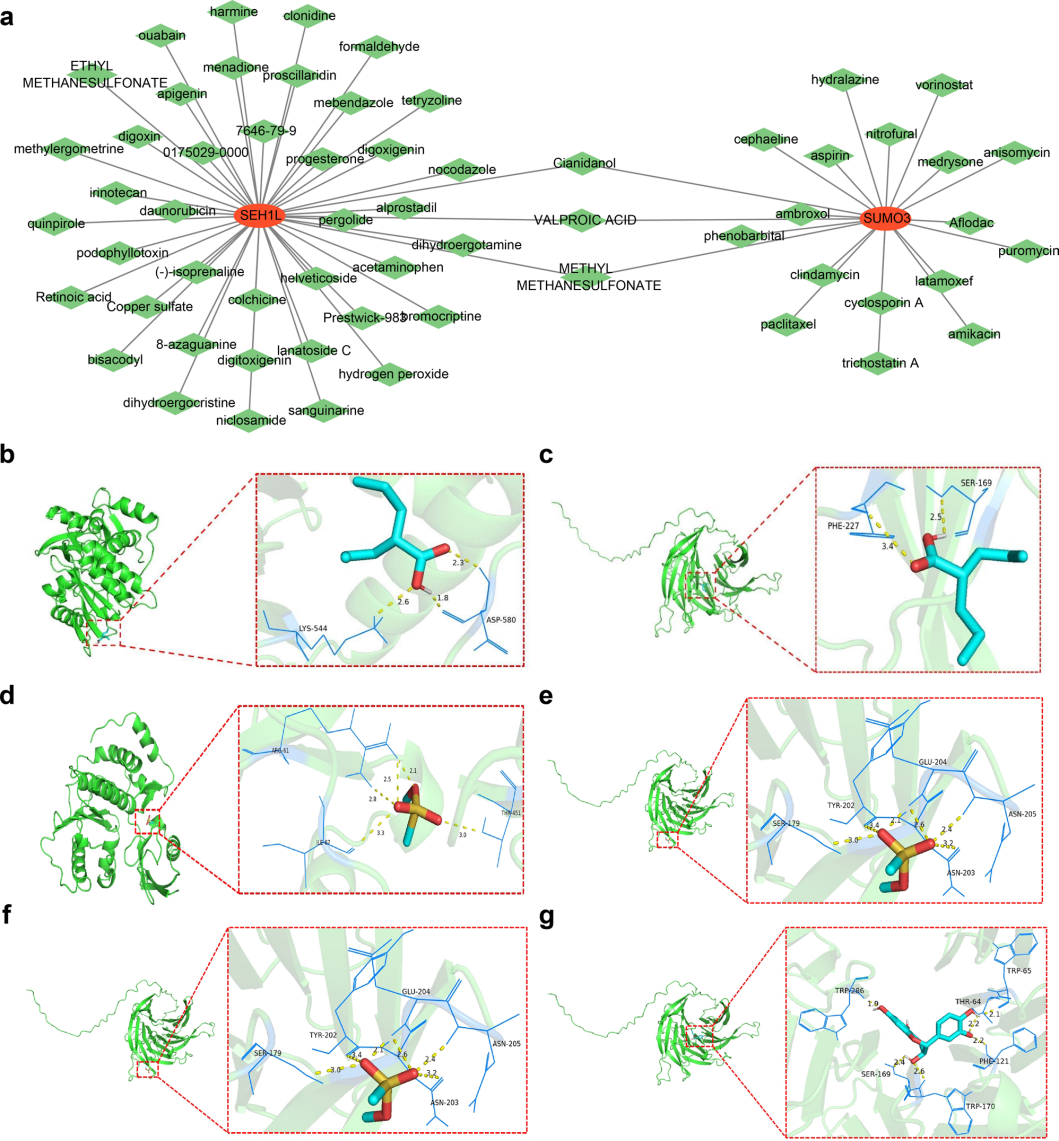
Gene-drug regulatory network and molecular docking. (**A**) Gene-drug regulatory network. Red nodes represent biomarkers, green nodes represent drugs. (**B**) Binding ability between Valproic Acid and the residue LYS-544 and ASP-580 of SUMO3. (**C**) Binding ability between Valproic Acid and the residues PHE-227 and SER-169 of SEH1L. (**D**-**E**) Binding ability between Methanesulfonate and SUMO3/ SEH1L. (**F**-**G**) Binding ability between Cianidanol and SUMO3/ SEH1L. Light blue and red double-ring stick models represent active molecules. Dark blue stick structures are amino acid residues with hydrogen bond interactions with active ingredients. Yellow dashed lines show hydrogen bonds between active ingredients and amino acid residues

Based on data from the DSigDB database, a total of 20 drugs were predicted for SUMO3, and 43 drugs for SEH1L. A drug-biomarker network comprising 62 nodes and 63 edges was constructed, highlighting interactions such as SEH1L-bromocriptine and SUMO3-paclitaxel (Fig. 9A). Notably, three drugs—Cianidanol, Methyl methanesulfonate (MMS), and Valproic Acid—were predicted to interact with both biomarkers. Molecular docking simulations were then performed to assess the binding affinity of these compounds with the biomarkers (Table 2). Valproic Acid formed a hydrogen bond with the residues LYS-544 and ASP-580 of SUMO3, with a binding energy of -4.3 kcal/mol (Fig. 9B). Similarly, Valproic Acid formed a hydrogen bond with PHE-227 and SER-169 of SEH1L, with a binding energy of -4.2 kcal/mol (Fig. 9C). The binding energies for MMS were − 3.8 kcal/mol for SUMO3 and − 3.3 kcal/mol for SEH1L (Fig. 9D-E). For Cianidanol, SUMO3 exhibited a binding energy of -7.8 kcal/mol, while SEH1L showed a slightly lower binding energy of -7.6 kcal/mol (Fig. 9F-G). These results suggest that Cianidanol could be a promising therapeutic candidate for targeting both biomarkers.

**Table 2:** Binding energy of molecular docking

Symbol	Molecule Name	PubChem CID	affinity(kcal/mol)	hydrogen bonds
SUMO3	Cianidanol	9064	-7.8	5
	Methyl methanesulfonate	4156	-3.8	5
	Valproic Acid	3121	-4.3	3
SEH1L	Cianidanol	9064	-7.6	6
	Methyl methanesulfonate	4156	-3.3	6
	Valproic Acid	3121	-4.2	2

### RT-qPCR of SUMO3 and SEH1L

**Fig. 10:**
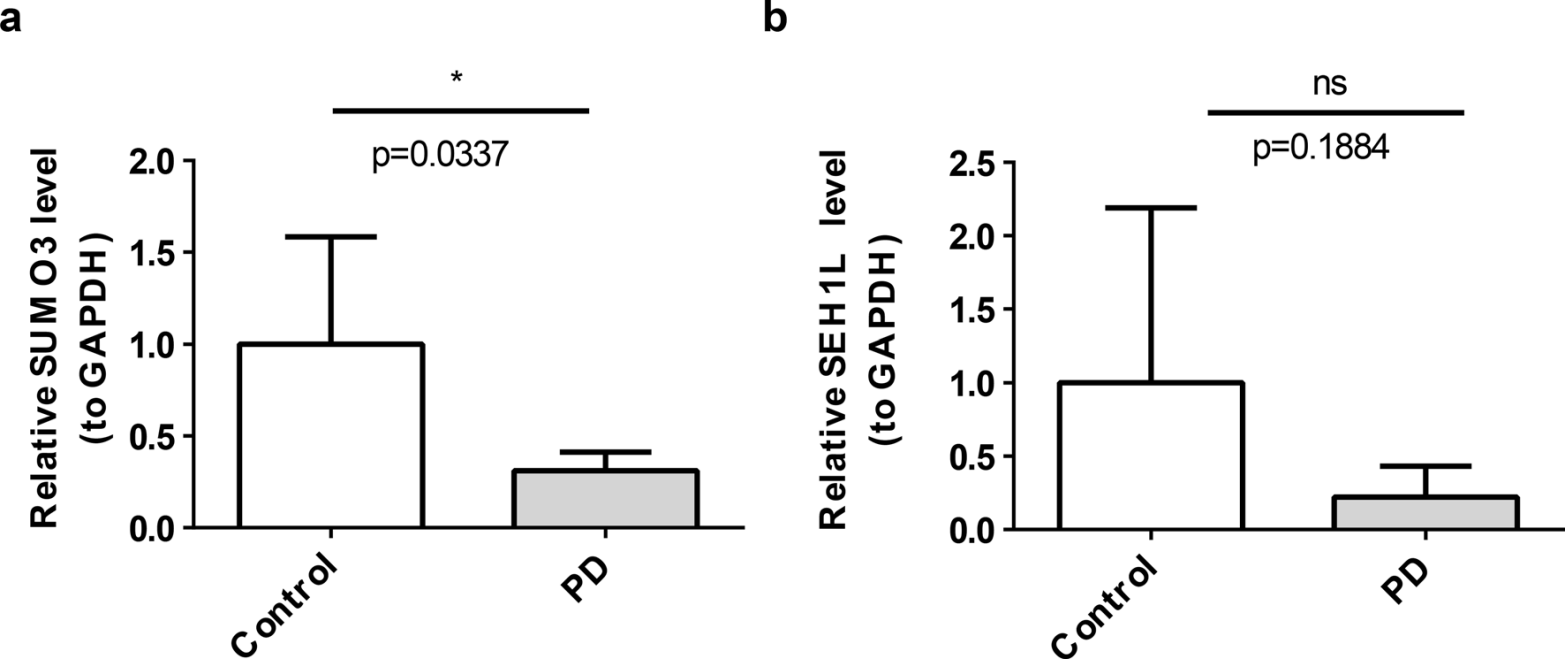
RT-qPCR. (**A**) RT-qPCR of SUMO3 in control and PD samples. (**B**) RT-qPCR of SEH1L in control and PD samples. *n* = 5 PD / 5 controls for RT-qPCR. ns represents not significant, * *P* < 0.05

Finally, RT-qPCR was conducted to validate the expression levels of SUMO3 and SEH1L in control and PD samples. The RT-qPCR results confirmed that SUMO3 expression was significantly down-regulated in PD samples (*P* = 0.0337), consistent with the bioinformatics analysis (Fig. 10A; Table 3). SEH1L exhibited a non-significant downregulation trend (*P* = 0.188) in PD samples, warranting further validation in larger cohorts (Fig. 10B; Table 3).

**Table 3:** RT-qPCR analysis

	Control	PD	*p*
SUMO3	1 ± 0.5836	0.3113 ± 0.1010	0.0337*
SEH1L	1 ± 1.1912	0.2222 ± 0.2098	0.1884

Note: 1.* P <0.05; 2. SEH1L downregulation was not statistically significant (P = 0.188). RT-qPCR, reverse transcription-quantitative polymerase chain reaction

## Discussion

PD is a prevalent neurodegenerative disorder [41], with the abnormal aggregation and transmission of α-synuclein in the substantia nigra playing a pivotal role in its onset and progression [42]. Sumoylation, a PTM, contributes to protein misfolding, aggregation, impaired degradation, and compromised clearance mechanisms, all of which ultimately lead to neuronal dysfunction and cell death [43]. Moreover, SUMOylation is intricately linked to the aberrant aggregation and degradation of α-syn oligomers. Despite its significance, comprehensive studies on SUMOylation in PD remained limited. Existing studies had demonstrated that Sumoylation played a crucial role in the pathogenesis of PD, particularly in relation to α-synuclein aggregation, the functional regulation of DJ-1, mitochondrial bioenergetics, and immune responses [43]. However, comprehensive analyses of SUMOylation in the context of PD remain relatively scarce, with current research predominantly focusing on processes such as phosphorylation and methylation [44, 45]. Moreover, existing studies have largely centered on the effects of individual SUMO-related proteins (e.g., SUMO1) on specific regulatory mechanisms, lacking in-depth exploration of the global functional network underlying SUMOylation [46]. In the present study, in contrast to previous PD research focusing on non-SUMO modifications or specific sites, we identified SUMO3 and SEH1L—both associated with SUMOylation—as potential novel biomarkers for PD. This was achieved through comprehensive bioinformatics analyses, employing machine learning approaches including LASSO regression, SVM-RFE, and the Boruta algorithm, coupled with validation across multiple datasets and RT-qPCR verification. On one hand, this discovery partially addressed the existing gap concerning the role of Sumoylation genes in PD, thereby offering a new avenue for research into both Sumoylation genes and PD. Furthermore, the identification of novel biomarkers carried significant implications for the diagnosis of PD, management of disease progression, and development of innovative therapeutic agents. Initially, DEGs between PD and control groups in the GSE22491 dataset were identified. A subsequent intersection with SUMO-RGs led to the selection of 25 candidate genes. GO enrichment analysis revealed that these genes were significantly enriched in processes such as chromosome condensation and intracellular receptor signaling pathways. Notably, structural chromosomopathies have frequently been associated with PD, with a particular link to 22q11.2 deletion syndrome [47]. Furthermore, dysregulation of the JAK/STAT signaling pathway, involving abnormal activation or phosphorylation of key components, has been implicated in PD pathogenesis [48]. KEGG pathway analysis identified 28 enriched pathways, including nucleocytoplasmic transport and the cell cycle. Disruptions in nucleocytoplasmic transport (NCT) have been shown to play a critical role in PD [49], while alterations in cell cycle regulation contribute to cellular senescence [50], premature aging, and apoptosis [51].

Following this, the LASSO and SVM-RFE algorithms were employed to refine the selection of biomarkers, highlighting SUMO3 and SEH1L as key candidates. Both in the GSE22491 and GSE18838 datasets, the expression levels of these biomarkers were found to be significantly reduced in PD samples. Notably, the AUC values for both SUMO3 and SEH1L exceeded 0.7 in both datasets, indicating strong diagnostic performance and supporting their potential utility as biomarkers for PD.SUMO3 and SEH1L were expected to serve as auxiliary indicators for the early diagnosis of PD and contribute to enhancing the accuracy and timeliness of PD diagnosis. Additionally, the current diagnosis of PD mainly depended on clinical symptoms, neuroimaging examinations, and the assessment of dopaminergic neuron function, etc. Nevertheless, in the early stage of PD, when the onset symptoms were not typical, these methods had certain limitations in diagnosis and monitoring of disease progression [52]. SUMO3 and SEH1L assisted in identifying potential patients at an early stage, enabling patients to receive treatment earlier and preventing the worsening of disease progression.

Variants in the SUMO3 gene are integral to the initiation of SUMOylation, a process essential for maintaining proper neuronal function [53]. SUMOylation has been shown to exert isoform- and site-specific effects on the aggregation of α-synuclein, emphasizing the protein’s vulnerability to post-translational modification by the SUMO [54]. DJ-1, a protein involved in PD, plays a critical role in regulating gene transcription and mitigating intracellular oxidative stress [55]. SUMOylation activates DJ-1, thereby enhancing the proteasomal degradation of mitochondrial outer membrane protein fission 1 (Fis1), a key mediator of mitochondrial fragmentation [56]. Variations in the SUMO3 gene can influence the pathological processes involving α-syn and DJ-1 post-translational modifications, both of which are central to the pathogenesis of PD [57]. Furthermore, two single nucleotide polymorphisms (SNPs) in the SUMO3 gene, rs180313 and rs235293, have been found to be significantly altered in patients with PD, suggesting their potential contribution to the pathophysiology of the disease [58]. Previous bioinformatics analyses have indicated a down-regulation of SUMO3 expression in patients with PD [59]. Similarly, studies have reported reduced levels of SUMO-modified α-syn in patients with PD [60], and our RT-qPCR experiments corroborated these observations, revealing consistent expression patterns for SUMO3 in both control and PD samples. Notably, SUMO3 expression was found to be significantly reduced in PD samples. These results underscore the potential of SUMO3 as a key biomarker in PD pathogenesis and highlight its promise as a therapeutic target [60].

The SEH1L encoded by the SEH1L gene, is crucial for mitosis, specifically for chromosomal binding during cell division [61]. SEH1L has emerged as a promising therapeutic target in various malignancies and other pathological conditions, and it plays a significant role in regulating the mechanistic target of rapamycin (mTOR) pathway in breast cancer [62]. Bioinformatics analyses have also identified SEH1L as a pivotal ubiquitin-related gene involved in immune infiltration in patients with osteoarthropathy [63]. Additionally, SEH1L is strongly associated with macrophage function [64] and has been implicated in the pathophysiology of epilepsy [52] and other neurodevelopmental disorders [65].

It is noteworthy that our study provides the first evidence of SEH1L’s involvement in the pathogenesis of PD. RT-qPCR analysis revealed a downward trend in SEH1L expression in PD samples, although the difference did not reach statistical significance. This may be attributed to the limited number of validation samples used in our study. It is well known that clinically manifested PD arises from complex interactions between genetic mutations and environmental factors, many of which exhibit incomplete penetrance [66]. Environmental factors can both protect against and increase susceptibility to the disease [67]. Together, these factors influence the clinical onset of PD.

In this study, GSEA was performed to explore the signaling pathways associated with SUMO3 and SEH1L. The results revealed that both SUMO3 and SEH1L were co-enriched in pathways related to snca and 26 S proteasome-mediated protein degradation. Notably, pathways such as Medicus Variant Mutation Inactivated Ubqln2 to 26 S Proteasome-Mediated Protein Degradation, Medicus Reference 26 S Proteasome-Mediated Protein Degradation, and Medicus Variant Mutation-Induced Aberrant SNCA to 26 S Proteasome-Mediated Protein Degradation were identified. SEH1L was found to be significantly associated with translation initiation and voltage-gated Ca^2+^ channel pathways, which are involved in calcium ion entry [68]. The 26 S proteasome, an archetype of ubiquitin-dependent proteasomes, is a barrel-shaped complex capable of degrading substrates with extended unstructured regions [69]. Recent studies have underscored the importance of 26 S proteasome-mediated protein degradation in the pathophysiology of PD. Additionally, SUMOylation of extranuclear proteins has been implicated in the regulation of neuronal and synaptic function [70], as well as in modulating synaptic transmission [71]. Research has also demonstrated that SUMO-mediated delayed α-synuclein degradation occurs through the proteasomal inhibitor MG132 and the lysosomal degradation inducer PMA [72].

Immune infiltration analysis revealed significant differences in the levels of five immune cell types between patients with PD and controls, including naive B cells, macrophages M0, resting mast cells, monocytes, and activated NK cells. Patients with PD often exhibit impaired CD4⁺ T cell function, a condition known as immunosenescence [73], and have elevated levels of circulating classical monocytes [74]. Bioinformatics analysis, including correlation and logistic regression analyses, highlighted mast cells as the most strongly associated with the onset of PD. In addition, two hub genes, RBM3 and AGTR1, were found to be linked to mast cells in the training cohort [75]. Another study employing single-sample gene set enrichment analysis (ssGSEA) identified three cuprotosis-related genes—ATP7A, SLC31A1, and DBT—that were correlated with immune cell activity and immune function in PD. This methodology could potentially offer more accurate diagnostic insights into PD progression and provide new therapeutic targets for patients with PD [76].

To identify potential drugs targeting the biomarkers, this study utilized the DSigDB database, which predicted several drugs, including Cianidanol, MMS, and valproic acid, as relevant for treatment. These drugs were selected for further molecular docking analysis. Cianidanol (25 µM) has been shown to protect neurons from toxicity and apoptosis induced by rotenone in differentiated SH-SY5Y cells and to improve motor and cognitive deficits in rotenone-induced rat models. These findings suggest that Cianidanol possesses neuroprotective effects in PD models and may represent a promising therapeutic candidate for PD treatment [77]. Although there is limited research on the use of valproic acid [78] and MMS [79] in PD therapy, our molecular docking analysis revealed that valproic acid forms hydrogen bonds with SUMO3 residues LYS-544 and ASP-580, as well as with SEH1L residues PHE-227 and SER-169. The binding energy of SUMO3 to MMS was calculated to be -3.8 kcal/mol, while the corresponding value for SEH1L was − 3.3 kcal/mol. These findings provide a solid foundation for exploring novel therapeutic applications of existing drugs. However, further validation through in vivo and in vitro experiments is required to substantiate these molecular docking results and their potential therapeutic implications.

At the level of disease diagnosis, the discovery of SUMO3 and SEH1L brings new opportunities for early diagnosis. SUMO3 is significantly downregulated in PD samples and demonstrates strong associations with PD across multiple dataset analyses, making it a crucial reference indicator for early diagnosis. Combined detection of SUMO3 and SEH1L expression levels may enhance the accuracy of early PD diagnosis, enabling early detection and intervention. Genetic variations in SUMO3 and their impact on the SUMOylation process [80] provide new targets and strategies for PD treatment. As for SEH1L, although its specific mechanisms in PD require further investigation, its involvement in nuclear pore function, tRNA transport, and other processes [81], as well as its role as a potential therapeutic target in various diseases, suggests that interventions targeting SEH1L could modulate related cellular functions and ameliorate PD pathology. In terms of drug development, the study predicted drugs that interact with SUMO3 and SEH1L, such as Cianidanol, Methyl methanesulfonate, and Valproic acid. Among these, Cianidanol has shown neuroprotective effects in cellular and animal models, improving motor and cognitive dysfunction [82]. These predicted drugs provide directions for developing targeted therapies for SUMO3 and SEH1L, which can be further validated through in vitro and in vivo experiments, opening new avenues for PD drug development.

This study has achieved certain results in exploring biomarkers and therapeutic research for PD, but it also has some limitations. SUMO3 was significantly downregulated as validated by qPCR, strongly supporting its potential as a biomarker and its critical role in PD pathogenesis. However, although SEH1L showed involvement in key processes and co-enrichment with SUMO3 in PD-related protein pathways through bioinformatics analysis, its qPCR results did not reach statistical significance. The small sample size and differences in sample types may be reasons for the insufficient evidence regarding SEH1L. On one hand, the limited sample size makes it difficult to fully encompass the complex genetic backgrounds and diverse clinical features of PD patients, affecting the accuracy of detecting SEH1L expression differences. On the other hand, this study used datasets based on peripheral blood mononuclear cells (GSE22491) and whole blood (GSE18838). Different sample types naturally exhibit differences in gene expression patterns due to variations in cell composition and characteristics, which may interfere with the accurate identification of key biomarkers. Furthermore, there is currently a paucity of precise experimental evidence to confirm the specific regulatory mechanisms between SUMO3, SEH1L, and PD, necessitating additional functional validation experiments.

Future research could expand in multiple directions. Firstly, increasing the sample size by including a broader range of PD patients with diverse genetic backgrounds and rich clinical features to enhance the reliability and generalizability of the results. For SEH1L, further qPCR validation should be conducted alongside techniques such as Western Blot and immunohistochemistry to confirm its expression changes at the protein level and its role in PD pathogenesis. Moreover, functional studies could be carried out, such as investigating the effects of SEH1L gene silencing or overexpression on cellular functions and PD-related pathological processes to clarify its reliability as a biomarker and its value as a potential therapeutic target. Furthermore, molecular mechanism research should be strengthened. In the future, we will employ multicenter, large-sample prospective cohorts, standardize sampling and RNA extraction protocols, and integrate single-cell sequencing technology to dissect the expression profiles of candidate genes in specific cardiovascular immune cell subsets. This strategy is designed to validate the cross-cell-type universality and clinical robustness of these candidate genes. For future investigations, reference may be made to the research conducted by Asadi, Abed, and their respective teams on ceRNAs, non-coding RNAs, and immune-related genes [83–85], with a focus on delving into the molecular mechanisms underlying PD at the level of disease molecular fundamentals. Furthermore, we can increase functional verification related to SUMO binding and study the effect of SUMOylation of SUMO3 and SEH1L on cell function, thereby providing a more solid theoretical basis for the early diagnosis and treatment of PD.

## Conclusions

In conclusion, through a series of comprehensive analyses—including differential expression analysis, PPI network construction, machine learning, expression level analysis, ROC analysis, and RT-qPCR validation—this study successfully identified two SUMO-related genes (SUMO3 and SEH1L) as potential biomarkers for PD. Furthermore, regulatory networks of gene-transcription factor and gene-microRNA, as well as association networks of gene-disease and gene-drug, were established. These findings suggest that SUMO3 and SEH1L could act as valuable potential biomarkers for PD, providing new therapeutic perspectives and potential drug targets for the clinical management of PD patients. However, further validation using larger sample sizes and additional experimental methods is necessary to confirm SEH1L’s suitability as a biomarker.

## Supplementary Information

Below is the link to the electronic supplementary material.

Supplementary Material 1

## Data Availability

The data that support the findings of this study are openly available in the [GEO] at [https://www.ncbi.nlm.nih.gov/gds], reference number [GSE22491, GSE18838, GSE6613] and the [MSigDB] at [https://www.gsea-msigdb.org/gsea/index.jsp].

## Abbreviations

PD: Parkinson’s disease
PTMs: protein post-translational modifications
SUMO: small ubiquitin-like modifier
SUMOylation: small ubiquitin-like modifier conjugation
SUMO-RGs: SUMOylation-related genes
DEGs: differentially expressed genes
α-syn: α-synuclein
DMV: dorsal motor nucleus of the vagus
LC: Locus Coeruleus
HD: Huntington’s disease
AD: Alzheimer’s diseas e
SVM-RFE: Support Vector Machine Recursive Feature Elimination
RT-qPCR: Reverse transcription-quantitative polymerase chain reaction
GO: Gene Ontology
KEGG: Kyoto Encyclopedia of Genes and Genomes
PPI: Protein-Protein Interaction
ROC: Receiver operating characteristic
AUC: Area Under Curve
FPD: fingerprint networks
BP: Back propagation
GSEA: Gene Set Enrichment Analysis
GSVA: Gene Set Variation Analysis
TFs: transcription factors
DSigDB: Database of Signatures
PDB: Protein Data Bank
CCs: Cellular Components
MFs: Molecular Functions
MMS: methyl methanesulfonate
SEH1L: SEH1-like nucleoporin
NK cells: natural killer cells
SNPs: single nucleotide polymorphisms
NCT: nucleocytoplasmic transport
