# Supplementary material for: Screening, identification, and experimental validation of SUMOylation biomarkers in Parkinson’s disease
Source: Hereditas. 2025 Aug 8;162:154. doi: 10.1186/s41065-025-00525-1 (PMC12335102; doi:10.1186/s41065-025-00525-1)
Supplement: Supplementary file 1 — Supplementary Material 1 [file 41065_2025_525_MOESM1_ESM.zip › Supplementary material/Supplementary_Material(figures and tables).docx]

Supplementary Material

# Supplementary Figures and Tables

## Supplementary Figures


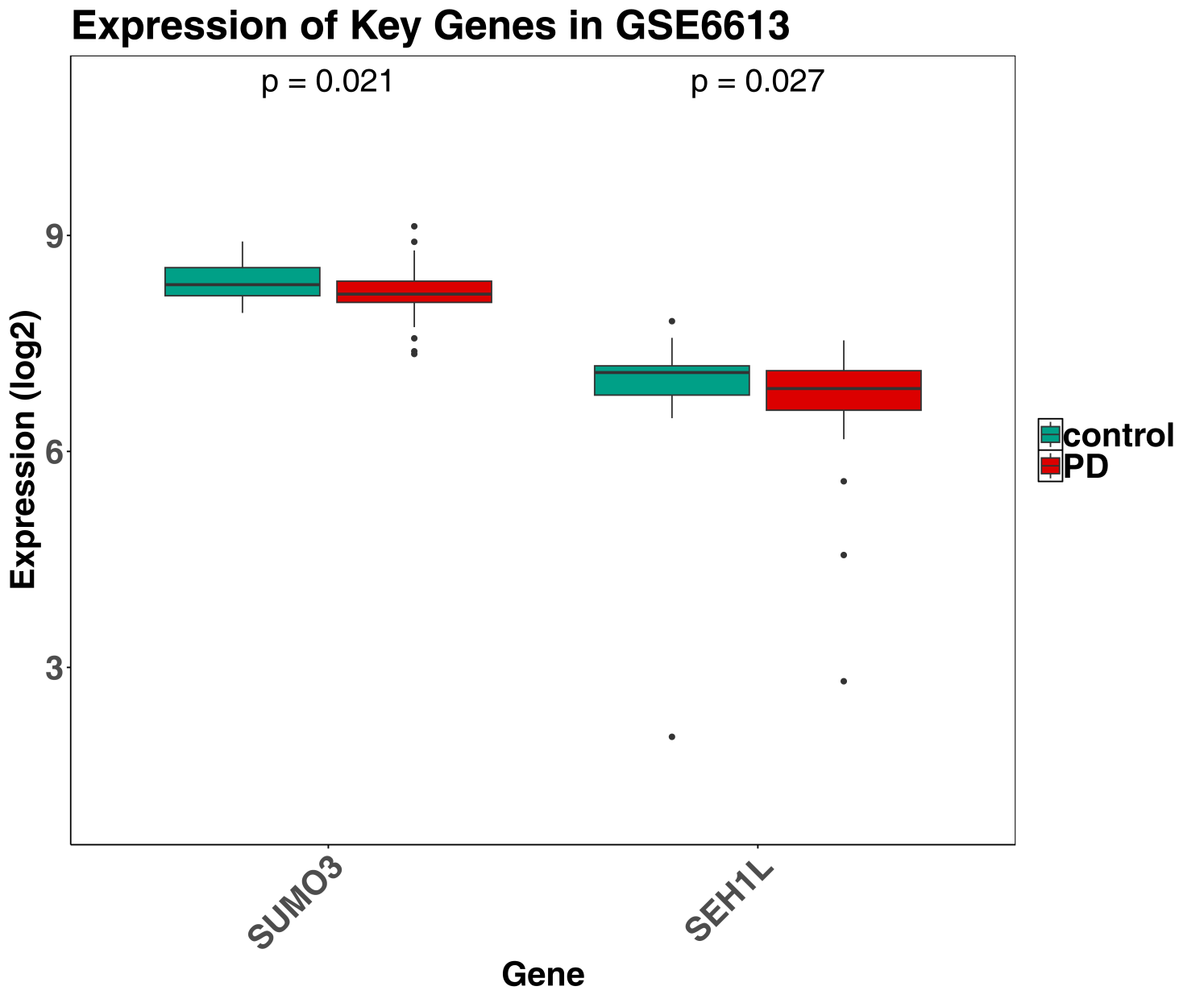


**Supplementary Figure 1.** The validation of the expression levels of biomarkers in the GSE6613 dataset.


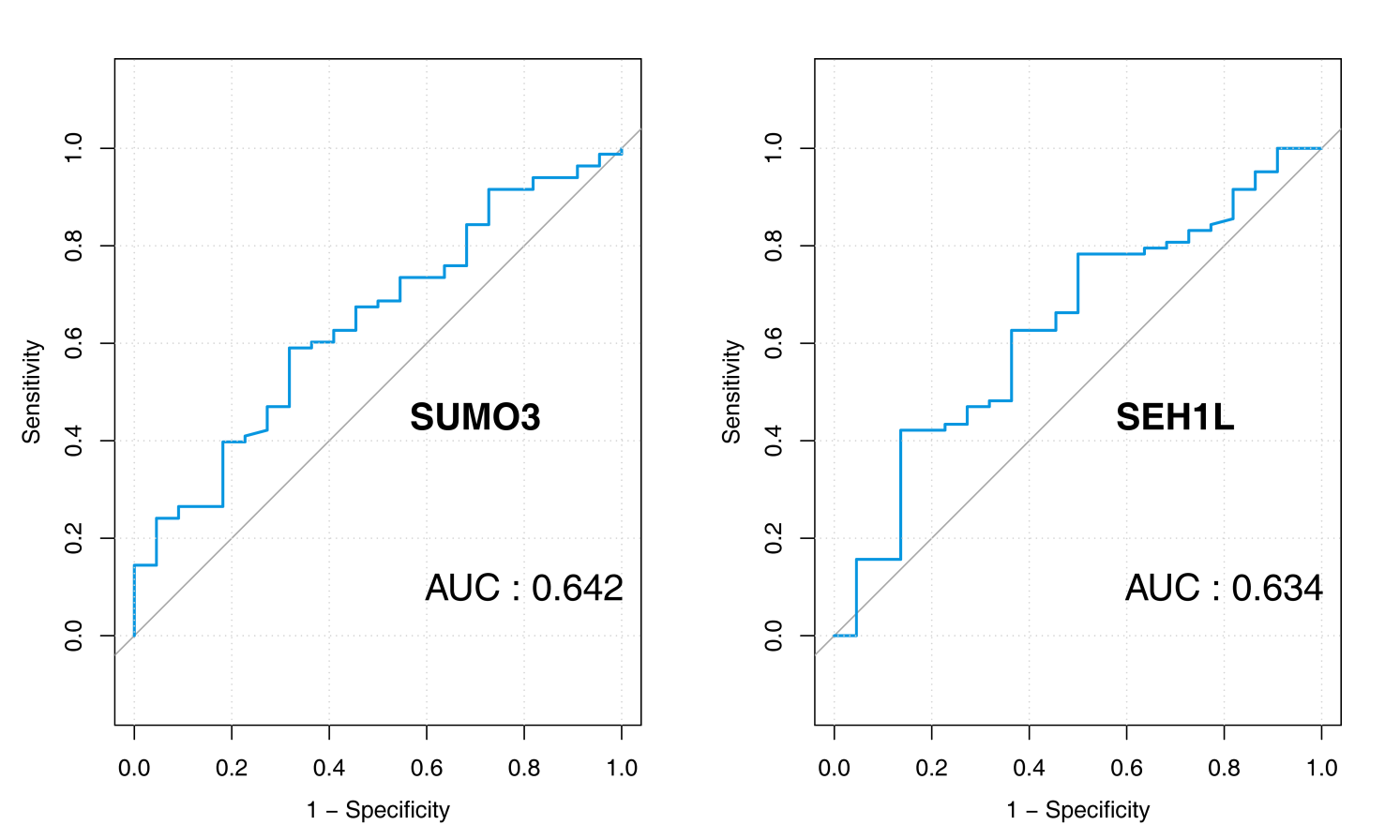


**Supplementary Figure 2：**The ROC curve of the GSE6613 dataset.

## Supplementary Table

**Supplementary Table 1:** The primer sequences of biomarkers.

| primer (5’-3’) | sequences | |
| --- | --- | --- |
| SUMO3-F | CAAGCTGATGAAGGCCTACTG | |
| SUMO3-R | TGTGCTGGAGTGTCAGTTTC |  |
| SEH1L-F | GAGCGTTAAGGTCTGGGATAAA |  |
| SEH1L-R | CATGTCACACGCCATACAGA |  |
| GAPDH-F | CGAAGGTGGAGTCAACGGATTT |  |
| GAPDH-R | ATGGGTGGAATCATATTGGAAC |  |

**Supplementary Table 2:** The version number of R package.

| R package | version number |
| --- | --- |
| R | v 4.2.3 |
| limma | v 3.58.1 |
| ggplot2 | v 3.4.4 |
| pheatmap | v 1.0.12 |
| VennDiagram | v 1.2.3 |
| clusterProfiler | v 4.10.0 |
| Cytoscape | v 3.10.0 |
| glmnet | v 4.1 - 8 |
| e1071 | v 1.7.13 |
| Boruta | v 8.0.0 |
| rstatix | v 0.7.2 |
| pROC | v 1.18.4 |
| RCircos | v 1.2.2 |
| neuralnet | v 1.44.2 |
| psych | v 2.2.9 |
| stats | v 4.3.2 |
| AutoDock Vina | v 1.1.2 |
| PyMol | v 2.5.2 |
