# Supplementary figures and images for: Screening, identification, and experimental validation of SUMOylation biomarkers in Parkinson’s disease
Source: Hereditas. 2025 Aug 8;162:154. doi: 10.1186/s41065-025-00525-1 (PMC12335102; doi:10.1186/s41065-025-00525-1)

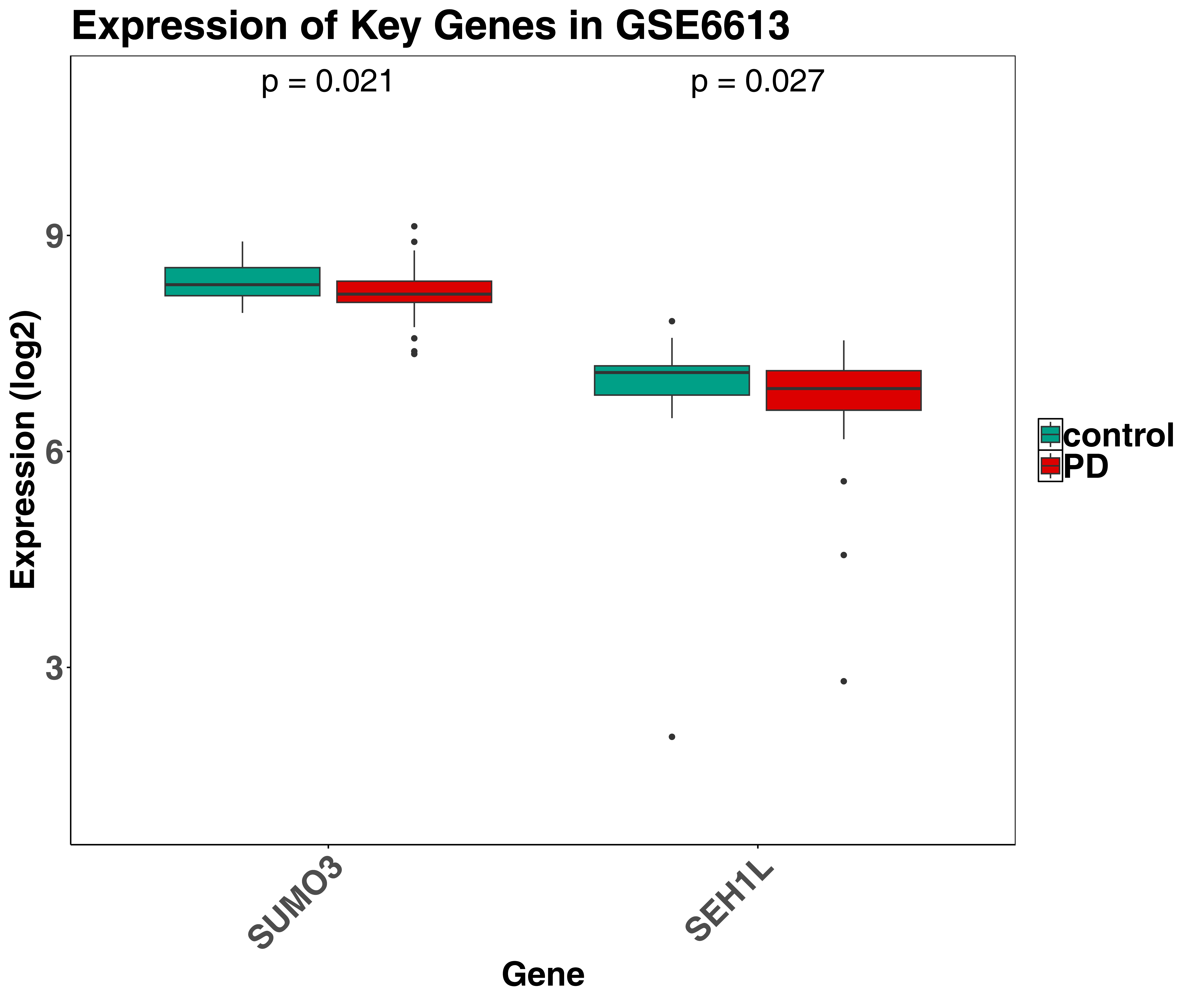

Supplement: Supplementary file 1 — Supplementary Material 1 [file 41065_2025_525_MOESM1_ESM.zip › Supplementary material/Supplementary Figure 1.tif]

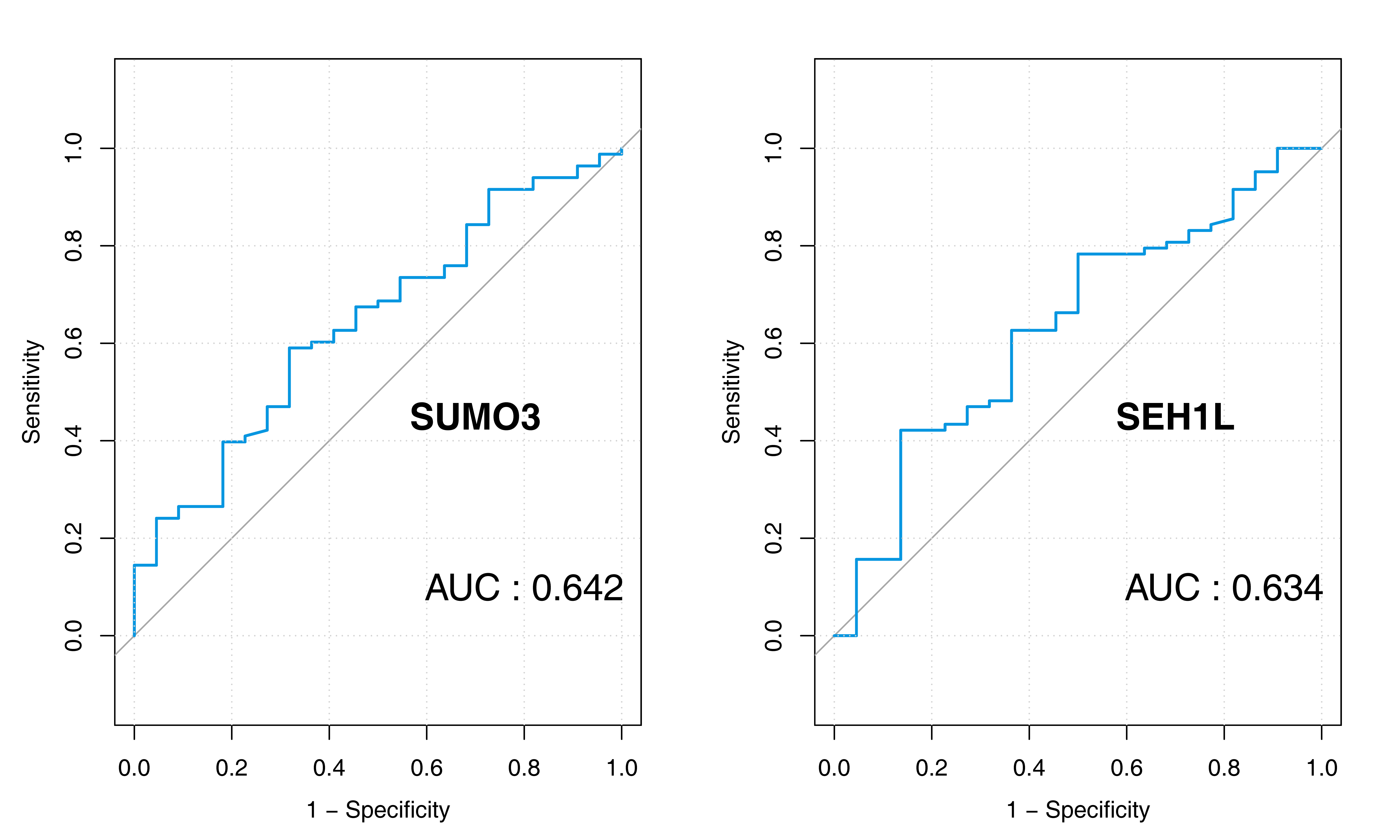

Supplement: Supplementary file 1 — Supplementary Material 1 [file 41065_2025_525_MOESM1_ESM.zip › Supplementary material/Supplementary Figure 2.tif]
